# Photoinduced Picosecond‐Scale Radial Current for Generating and Converting Spin Textures

**DOI:** 10.1002/nap2.70060

**Published:** 2026-04-04

**Authors:** Xiaoqiang Zhang, Pierre Vallobra, Yong Xu, Xiaoyang Lin, Weisheng Zhao

**Affiliations:** ^1^ State Key Laboratory of Spintronics Hangzhou International Innovation Institute Beihang University Hangzhou China

**Keywords:** heavy metal/ferromagnetic heterostructure, photoinduced, radial current, spin textures, spin–orbit torque

## Abstract

Magnetic skyrmions are topologically protected spin textures characterized by their whirling structure, high stability, and potential for next‐generation magnetic devices. However, their practical implementation has been hindered by the absence of methods for ultrafast and energy‐efficient skyrmion writing and erasure. We theoretically propose the generation of a picosecond‐scale photoinduced radial current that produces an azimuthally polarized spin–orbit torque in a heavy‐metal/ferromagnet heterostructure. This approach enables ultrafast (< 50 ps) generation and controlled transitions between four distinct types of topological skyrmionic quasiparticles. Our results establish a versatile platform for optically controlled topological magnetism and open pathways for high‐speed, low‐power spin‐based memory and logic technologies.

## Graphical abstract

A picosecond‐scale vector electrical current is generated and applied to generate and convert spin textures. Four target quasiparticle types with different topological states are generated with ultrafast speed (< 50 ps). Besides, the controlled transitions between these spin textures are achieved.

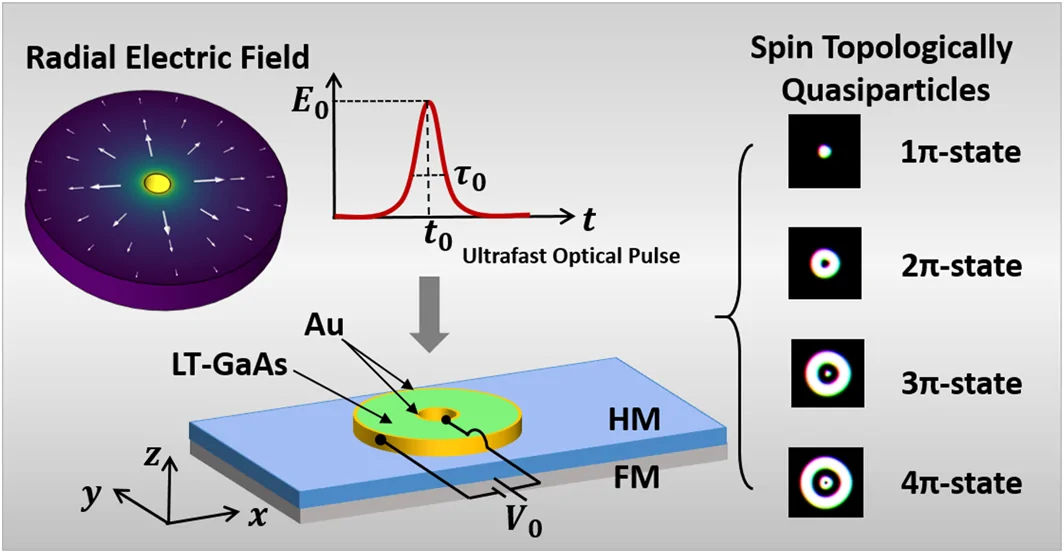

## 1 Introduction

With the impending end of Moore's law, alternative computing architectures demand urgent exploration. In particular, magnetic devices allow for reduced energy consumption since they are non‐volatile [1, 2, 3, 4]. Magnetic skyrmions, owing to their topological protection, are promising candidates for the next‐generation of magnetic devices, as they exhibit long‐term thermal stability even at the nanoscale [5, 6, 7]. Magnetic skyrmions are quasiparticles that possess a whirling magnetic texture that can be stabilized by the Dzyaloshinskii‐Moriya interaction (DMI) [8, 9]. Such interaction can only be present in a system with broken inversion symmetry and a high spin–orbit coupling. This was first realized in non‐centrosymmetric B20 crystals hosting Bloch skyrmions [10, 11]. In magnetic ultrathin films, Néel skyrmions can be stabilized due to the existence of an interfacial DMI [12]. Thin film skyrmions have been heavily scrutinized since they could replace the domain wall in racetrack memories to allow for higher stability and energy efficiency [5, 12]. However, the skyrmion Hall effect, a trajectory deflection arising from their non‐zero topological charge (*Q*), limits their utility [13, 14]. Two principal approaches have been developed to eliminate the topological charge and its associated skyrmion Hall effect [15]. The first method employs synthetic antiferromagnetic structures, where two ferromagnetic layers host skyrmions with opposite *Q* values that mutually cancel [16, 17]. The second strategy focuses on stabilizing alternative skyrmionic textures with intrinsically vanishing *Q* within a single ferromagnetic layer [9, 18]. The skyrmionium [9] (or named 2π quasiparticle, shortly written 2π QP in this paper) represents the simplest realization of this approach, exhibiting zero net *Q* and consequently no skyrmion Hall effect. More generally, any *n*π QP (where *n* is an integer) possesses vanishing topological charge when *n* is even [5]. Our recent work has extended this concept by demonstrating the stabilization of 4π QPs, which we found to achieve higher velocities than their 2π counterparts [19].

Although conventional skyrmions can be readily nucleated through magnetic fields [20, 21], electrical currents/voltages [22, 23], or optical excitation [19, 24], generating skyrmionium [9, 25], especially generating higher‐order *n*π QPs is a challenge. It requires more sophisticated control of spin dynamics, and little research has been reported. Recently, we theoretically propose the generation of a 4π QP using a light‐induced radially polarized magnetic field [19, 26]. However, due to the low magneto‐optical interaction, a very high pump laser fluence is necessary. Besides, since the laser pulse is on the picosecond timescale, the relaxation and stabilization into a topological spin texture requires several nanoseconds. Current induced generation and motion of spin textures have been studied for many years. Conventional current‐pulse techniques, limited by electronic bandwidth, have been first confined to the nanosecond‐to‐microsecond regime, hindering the observation of ultrafast spin dynamics. The recent development of picosecond‐scale electrical pulses has opened the door to studying ultrafast magnetic phenomena, including magnetization reversal and spin–orbit torque (SOT) switching [27, 28, 29]. However, this technology has so far only been used to generate traditional charge current, where it flows along a constant direction and it is a scalar. This approach could instead be leveraged to create vector ultrafast currents with complex geometries, such as radial or azimuthal flow [30, 31, 32]. In 2019, Göbel et al. surveyed optoelectrical writing and deleting mechanisms for skyrmioniums under a radially symmetric current pulse [33]. Although the authors utilized this geometry to produce skyrmionium, they overlooked the inherent current density gradient, a feature that could enable the generation of higher‐order quasiparticles [19].

In this work, we theoretically propose the generation of an ultrafast radial current that induces an azimuthally polarized SOT in an adjacent heavy metal/ferromagnetic (HM/FM) heterostructure. To achieve this, we propose a novel device architecture based on a low‐temperature‐grown GaAs (LT‐GaAs) photoconductive layer. Micromagnetic simulations reveal that a focused 50‐fs laser pulse under a radial bias voltage generates a picosecond radial photocurrent in the LT‐GaAs [28, 34], which in turn creates an azimuthally polarized SOT in the HM/FM bilayer. This unique torque geometry enables the ultrafast (< 50 ps) generation of all four quasiparticle types and facilitates controlled transitions between their topological states.

## 2 Picosecond Radial Direction Pulse Generation

We consider the schematic configuration shown in Figure 1a. A patterned structure, consisting of a 10 nm‐thick Pt layer (HM) and a 30 nm‐thick photoconductive LT‐GaAs ring, is stacked on a 1 nm‐thick FM layer, with two concentric annular Au electrodes deposited on the inner and outer edges of the LT‐GaAs ring, respectively. The LT‐GaAs ring can be fabricated by molecular beam epitaxy [27]. As shown in Figure 1b, the LT‐GaAs ring structure has inner and outer radii of 10 and 100 nm, respectively. Optical excitation is provided by a 50 fs laser pulse at a wavelength of 800 nm, focused onto the surface of the photoconductive LT‐GaAs. Meanwhile, a voltage of *V*_0_ = 1 V is applied between the two Au electrodes. A radial electric field pointing from the inner to the outer electrode (see Supporting Information S1: Figure S1) is established in the LT‐GaAs by applying a positive voltage to the inner Au electrode and a negative voltage to the outer one, as illustrated in Figure 1a. Such an electrically biased LT‐GaAs ring can generate picosecond currents to radiate terahertz (THz) waves due to its very short carrier lifetime and high carrier mobility [35]. Because the pump photon energy is greater than the bandgap of the LT‐GaAs, a large number of electron‐hole pairs are formed at the surface of LT‐GaAs. Given the thinness of the LT‐GaAs layer (30 nm), we neglect the attenuation of the pump beam and assume a uniform distribution of photogenerated electron‐hole pairs along the propagation direction. These photogenerated carriers are then accelerated by the built‐in radial electric field toward their respective Au electrodes: electrons drift toward the inner anode, and holes toward the outer cathode, as shown in Figure 1b. As a result, an ultrafast radial photocurrent pulse flows through the whole LT‐GaAs. To model the radial photocurrent pulse, we employ COMSOL Multiphysics [36, 37]. The electric field of the 50‐fs pump laser pulse, with a pulse delay of *t*_0_ = 0.5 ps, can be written as E(t)=E0e−t−t02τ2 as shown in the upper inset of Figure 1a. The pump laser peak intensity is *I*_0_ = 1 W/μm^2^, corresponding to a laser peak electric field *E*_*0*_ = 27.45 MV/m and a laser fluence *F* = 2.93 uJ/cm^2^. The electron and hole mobilities are 200 and 27 cm^2^/Vs, respectively. Their carrier lifetime is 100 and 400 fs, respectively [36].

**FIGURE 1:**
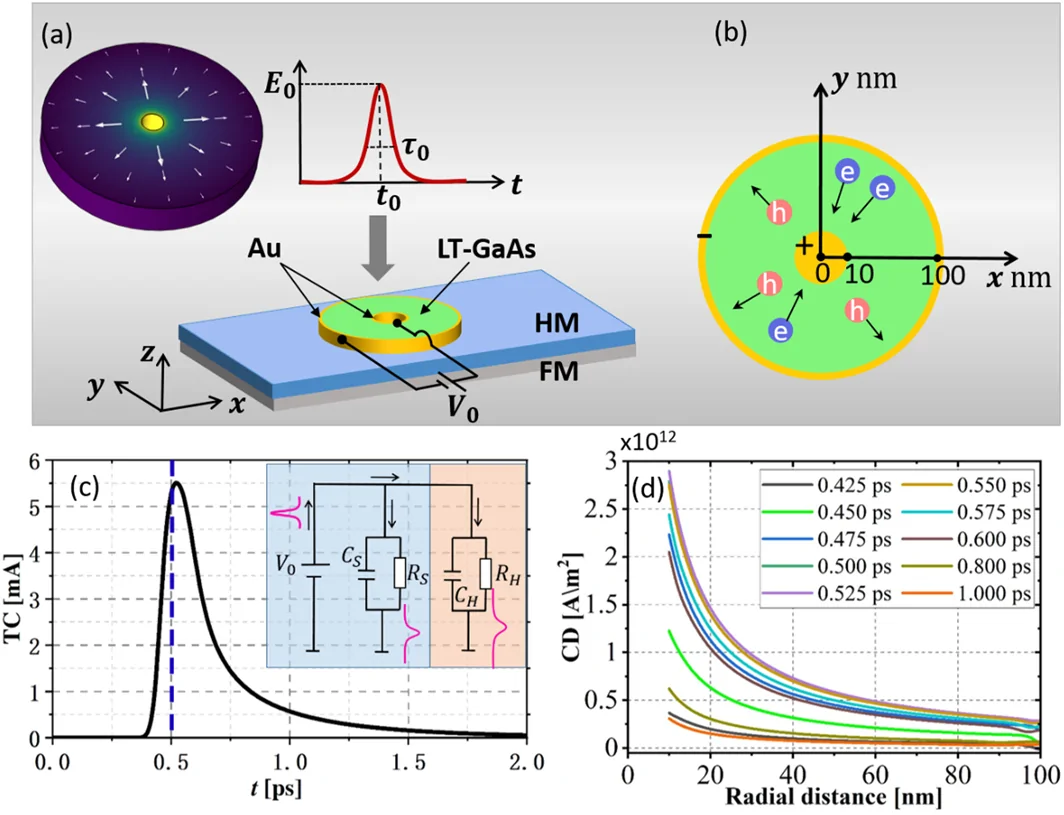
Schematic of photoinduced radial current. (a) Sketch of the geometry of the sample consisting of a multilayer stack of LT‐GaAs, HM, and FM. A laser pulse is irradiated on the ring LT‐GaAs layer, which is configured in a radially biased voltage *V*_0_. Two concentric annular Au electrodes are deposited on the inner and outer of the ring LT‐GaAs layer, respectively. The up inset shows the pump laser pulse with pulse duration *τ* = 50 fs and a pulse delay *t*_0_ = 0.5 ps. The left inset shows the calculated photoinduced current at *t* = 0.525 ps. (b) Geometry of the ring LT‐GaAs. The radius of the inner and the outer ring are 10 and 100 nm, respectively. Under the radially biased voltage, generated electrons *e* and holes *h* are accelerated toward respective Au electrodes. (c) Detected ultrafast radial photocurrent pulse flow through the two‐terminal Au electrodes. The inset shows the equivalent circuit of the proposed multilayer stack sample. (d) Distribution of the ultrafast radial current density (CD) along the *x*‐axis at different times.

As shown in Figure 1c, the terminal current (TC) generated by the ultrafast radial photocurrent features a sharp rise time and a prolonged decay tail, contrasting with the symmetric Gaussian shape of the excitation laser pulse and the previous hypothesis of a Gaussian photocurrent [33]. Besides, a time delay of about 250 fs exists between the peaks of these two pulses. We model the multilayer stack sample system using the equivalent circuit as shown in the inset of Figure 1c. The equivalent circuit with a blue background (left) models the LT‐GaAs layer, whereas the circuit with an orange background (right) represents the rest of the heterostructure. Both of them are represented as a parallel combination of resistances and capacitances [38]. Under laser excitation, the photoinduced ultrafast radial current first charges the capacitor Cs. This capacitor subsequently discharges through the resistance Rs, mimicking the behavior of an RC delay line [39]. It should be noted that Cs and Rs are dynamically evolving in response to changes in the density of photoinduced carriers (*n*_*h*_ and *n*_*e*_). As evidenced in Figure 1b, both types of carriers move along the radial direction. The left inset of Figure 1a shows the distribution of the photoinduced current *J* at *t* = 0.525 ps, where $J=en_{h}v_{h}-en_{e}v_{e}$ [36]. Here, *v*_*h*_ and *v*_*e*_ are the respective velocities of the induced hole and electron, and *e* is the charge of a proton. Figure 1d shows the photoinduced ultrafast radial photocurrent density along the *x*‐axis at different times. The associated current density decays exponentially along the radius. In the time domain, it starts to rise at *t* = 0.40 ps until it reaches its maximum value with current density (CD) of 2.8 × 10^12^ A/m at *t* = 0.525 ps for *x* = 10 nm. The current density then declines, becoming negligible after *t* = 1.6 ps.

By tuning the energy of the pump laser and the radially biased voltage, the photoinduced radial current density can be modified as discussed in Supporting Information S1: Note S2. As shown in Supporting Information S1: Figure S2, the current density initially increases with laser fluence before saturating at higher energy densities. To increase it further, we can select a larger biased voltage. Supporting Information S1: Figure S2 shows that the current density increases by more than 80 times, from 0.02 to 1.6, when the laser fluence and electric field strength are varied from *F*_*0*_ and *E*_*0*_ to 6*F*_*0*_ and 10*E*_*0*_, respectively. Here, *F*_*0*_ and *E*_*0*_ are the relative laser fluence and electric field strength [40]. We should point out that we apply a simple model to analyze the influencing factors of current density, which is an idealized linear response. In fact, in reality it likely breaks down. Besides, the characteristics of the LT‐GaAs, for example its band gap, the mobility and lifetime of generated carriers, can also affect the generation of the radial charge current. These properties are ultimately determined by growth conditions, which are beyond the scope of the present analysis. For the convenience of the subsequent calculations, we introduce a reference charge current density *J*_0_(*r*, *t*), induced by a pump laser peak intensity of *I*_0_ = 1 W/μm^2^ and a bias voltage of *V*_0_ = 1 V.

## 3 Effect of the Ultrafast Radial Current on the Magnetization

The radial charge current generated in the LT‐GaAs will also flow through the heterostructure below, as represented in the equivalent circuit of the proposed multilayer stack sample in Figure 1c. This current generates a SOT with an azimuthal polarization vector $\sigma =e_{\theta }$ [41, 42]. The distribution of the charge current in the ultrathin underlying heterostructure differs slightly from that in the upper LT‐GaAs layer due to the impedance associated with the couple capacitor *C*_H_—resistance *R*_H_. Although this impedance can be tuned—for example, by modifying geometric parameters such as thickness or radius—to achieve impedance matching [26, 28, 43], we neglect this difference in our model to simplify the analysis.

We simulate the effect of the photoinduced SOT on the FM layer using the software MuMax3. The SOT generated in the adjacent Pt layer comprises two components: a field‐like torque ($\tau_{SOT\_FL}$) and a damping‐like torque ($\tau_{SOT\_DL}$). We consider a 512 nm × 512 nm × 1 nm^3^ FM layer with magnetic parameters similar to our previous publication [19]. The time evolution of the magnetization **M** is calculated based on the Landau‐Lifshitz‐Gilbert (LLG) equation [9, 19]:

(1) $$\frac{dM}{dt}=-\gamma M\times H_{\text{eff}}+\frac{\alpha }{M_{s}}\;\left(M\times \frac{dM}{dt}\right)+\tau_{\text{SO}T\_\text{FL}}+\tau_{\text{SO}T\_\text{DL}}.$$

Here, *α*, *M*_*s*_, *γ*, and **H**_eff_ correspond to the Gilbert damping, the saturation magnetization, the gyromagnetic ratio and effective field, respectively. The effective field derives from the energy density,

(2) $$H_{\text{eff}}=\frac{1}{\mu_{0}}\frac{\partial \varepsilon }{\partial M}.$$

The effective field governing the magnetization dynamics comprises the Heisenberg exchange interaction, magnetic anisotropy, DMI and dipolar field and is given by:

(3) $$\varepsilon =A_{\text{ex}}{\;\left[\nabla \left(\frac{M}{M_{s}}\right)\right]}^{2}-K_{u\;}\frac{{\left(e_{k}\cdot M\right)}^{2}}{{M_{s}}^{2}}-\frac{\mu_{0}}{2}M\cdot H_{d}\left(M\right)+\frac{D}{{M_{s}}^{2}}\;\left(M_{z}\frac{\partial M_{x}}{\partial x}+M_{z}\frac{\partial M_{y}}{\partial y}-M_{x}\frac{\partial M_{z}}{\partial x}-M_{y}\frac{\partial M_{z}}{\partial y}\right).$$

In Equation (3), $A_{\text{ex}}$, $K_{u}$, and D denote the Heisenberg exchange energy constant, perpendicular magnetic anisotropy, and DMI energy constant, respectively. The perpendicular anisotropy unit vector and the demagnetization field vector are represented by **e**_*k*_ and **H**_d_(**M**), respectively. The material parameters used in this study are: *M*_*s*_ = 5 × 10^5^ A/m, $A_{\text{ex}}$ = 9.7 × 10^−12^ J/m, *D* = 3.0 × 10^−3^ J/m^2^, $K_{u}$ = 0.8 × 10^6^ J/m^3^, α = 0.1 [40]. The two SOT torques are expressed as [41]:

(4) $$\tau_{\text{SOT}\_\text{DL}}=\frac{u_{\text{SOT}}}{{M_{s}}^{2}d}\;\left(M\times \sigma \times M\;\right),$$

(5) $$\tau_{\text{SOT}\_\text{FL}}=\frac{\beta_{\text{SOT}}u_{\text{SOT}}}{M_{s}d}\;\left(M\times \sigma \;\right),$$

with $u_{\text{SOT}}$ = *γ*(ℏ*jα*_*H*_/2*eM*_s_). *d* denotes the film thickness in meters, *β*_SOT_ is the unitless non‐adiabaticity factor associated with SOT and *α*_H_ is the unitless current conversion factor. We assume *α*_*H*_ = 0.15 and $\beta_{\text{SOT}}$ = −2, consistent with previous reports on similar systems [44].

Figure 2 illustrates the evolution of the magnetization under varying current densities ranging from 12 × *J*_0_(*r*, *t*) to 46 × *J*_0_(*r*, *t*). We observe the formation of *n*π QPs, with *n* ranging from 1 to 4, whose emergence depends on the current amplitude. As expected from the cylindrical LT‐GaAs geometry, all magnetic configurations exhibit cylindrical symmetry. A comparison of the final states displayed on Figure 2b,g,l,r, reveals that the out‐of‐plane magnetization component (*m*_*z*_) increases with current density. We attribute this enhancement to the field‐like torque component of the SOT, which along the radial direction $e_{r}$, drives precessional dynamics around the azimuthal direction $e_{\theta }$. Concurrently, concentric rings emerge after the pulse to minimize DMI energy (Figure 2c,h,m,s). It can also be observed that the center of the beam retains its initial magnetization due to the absence of LT‐GaAs. These concentric rings subsequently relax into topologically stable quasiparticles, as shown in the equilibrium states (Figure 2e,j,o,u).

**FIGURE 2:**
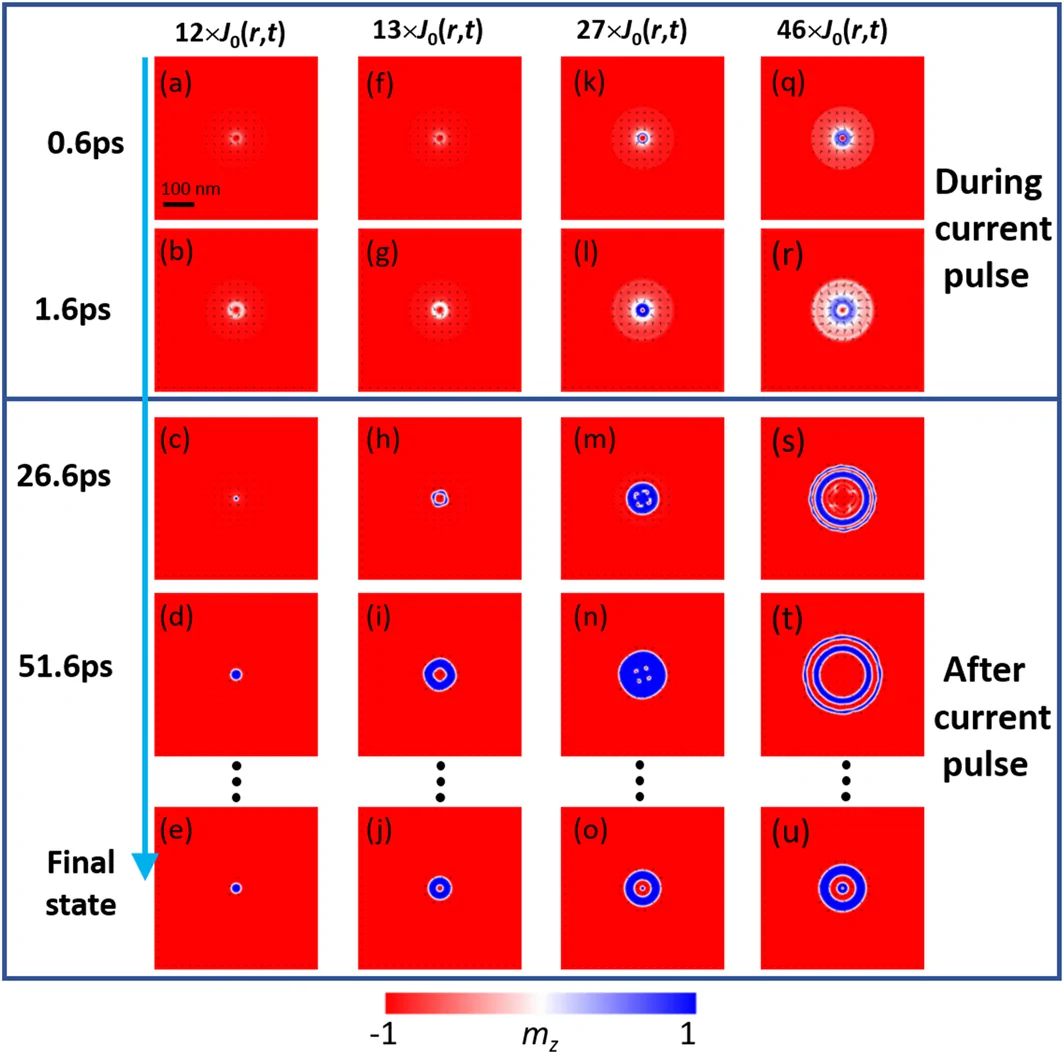
Generation of *n*π quasiparticles. Magnetization states at different time for a current density of 12 × *J*_0_(*r*, *t*) (a)–(e), 13 × *J*_0_(*r*, *t*) (f)–(j), 27 × *J*_0_(*r*, *t*) (k)–(o), and 46 × *J*_0_(*r*, *t*) (q)–(u), respectively. The final state is defined as the state where the magnetization stops evolving. The time to attain the final state depends on the initial size of the quasiparticles at the end of the pulse and the size of the stable quasiparticle.

Although the magnetic configurations at 51.6 ps differ from their final equilibrium states for each current density, all QPs retain their topology (i.e., π, 2π, 3π, or 4π) throughout the relaxation process. After this time, the QPs evolve primarily in size, shrinking to reach their equilibrium dimensions. Intriguingly, the size of the 4π QP after 51.6 ps is larger than the size of the beam. This reveals that the relaxation process of the 4π QP after the pulse first involves an initial expansion phase, similar to a breathing mode triggered by out of equilibrium dynamics. To quantitatively determine the quasiparticle generation time, we analyze the evolution of the topological charge *Q*, defined as $Q=\frac{1}{4\pi }\iint m\cdot \left(\frac{\partial m}{\partial x}\times \frac{\partial m}{\partial y}\right)dxdy$, where $m=M/M_{s}$ is the normalized magnetization [12, 19]. Figure 3a displays the time evolution of *Q* during the generation of the four quasiparticles shown in Figure 2.

**FIGURE 3:**
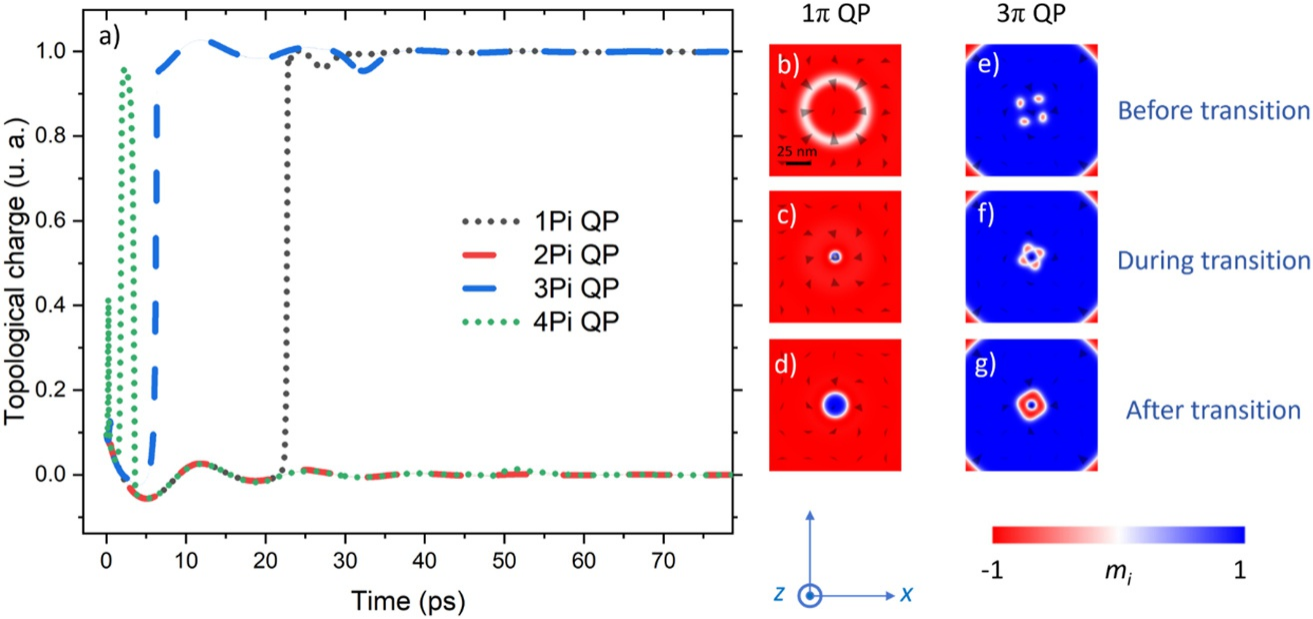
Topological transition of the quasiparticles. (a) Time evolution of the topological charge *Q*. Magnetic configuration of a 1π QP, (b) before, (c) during, and (d) after the topological transition. Magnetic configuration of a 3π QP, (e) before, (f) during, and (g) after the topological transition.

The evolution of the topological charge *Q* (Figure 3a) reveals distinct dynamics: whereas the 2π and 4π QPs show minimal variation in *Q*, the skyrmion (ππ QP) and the 3π QP each undergo a single, abrupt transition. These jumps in *Q* correspond to the creation or annihilation of a ring within the magnetic texture. To clarify these dynamics, we show the magnetic configurations evolution leading to the formation of the 1π QP in Figure 3b–d and of the 3π QP in Figure 3e–g. For clarity, these images display only the 128 nm × 128 nm square at the center of the pulse and use a different scale than Figure 2. The dynamical process can be found in Video S1 and S2. As previously observed in Figure 2b, a ring of in‐plane magnetization forms immediately after the pulse. This ring contracts radially until it reaches a critical size below which its topology is unstable, collapsing into the nanoscale skyrmion in Figure 3c. This event corresponds to the jump in *Q* observed at 25 ps in Figure 3a. The newly created skyrmion then expands to reach its equilibrium size, as observed in Figure 3d. A similar process of ring stabilization at the center, which occurs in Figure 3e–g and is associated with the jump of topological charge at 7 ps leads to the stabilization of the 3π QP. For both π QP and 3π QP, the change of topology can only occur because the energy of the system is initially higher than the potential barrier separating the topological states. This is a purely dynamical process; equilibrium energy landscapes alone cannot predict whether the final state will be a π, 2π, 3π, or 4π QP. In contrast, a transition from a 5π QP to a 4π QP can be explained by equilibrium energetics, since the 5π configuration lacks a local energy minimum in this material.

The topological charge *Q* stabilizes for all quasiparticles within 50 ps, establishing an upper bound for the generation time. These sub‐50 ps stabilization dynamics are governed by the competing contributions of the SOT components. To isolate their individual effects, Figure 4a–c displays the magnetic configuration at the end of the current pulse when only DL torque $\tau_{SOT\_DL}$ is present. Conversely, Figure 4d–f shows the configuration resulting solely from the field‐like torque ($\tau_{SOT\_FL}$).

**FIGURE 4:**
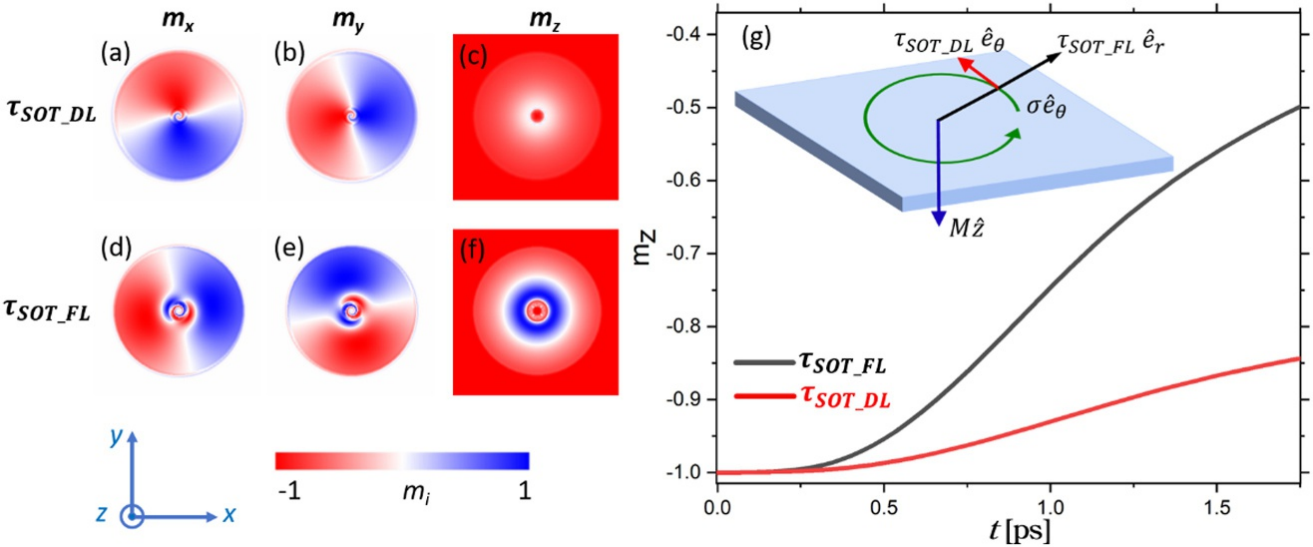
Effect of each component of the SOT on the magnetization. (a) *x*, (b) *y*, and (c) *z* components of the magnetic configuration at the end of the charge current pulse when only DL torque is present. (d) *x*, (e) *y*, and (f) *z* components of the magnetic configuration at the end of the charge current pulse when only FL torque is present. (g) Evolution of the magnetization in the area exposed to the pulse depending on the torque applied. The inset shows the direction of FL and DL torques.

As observed in Figure 4a,b, the DL torque promotes a stronger *m*_*x*_ component in the *y* direction and *m*_*y*_ component along the *x* direction. This is consistent with its mathematical form, which favors alignment of the magnetic moment along the azimuthal direction $e_{\theta }$ (see inset Figure 4g), parallel to the polarization vector. This tendency is further evidenced by the white ring in Figure 4c, indicating a region where the out‐of‐plane component *m*_*z*_ vanishes. In contrast, the FL torque induces a stronger *m*_*x*_ component in the *x* direction and *m*_*y*_ component in the *y* direction (Figure 4d,e). Given the ultrashort pulse duration, the main effect of the FL torque is to initiate precession around $e_{\theta }$ and in the *xOy* plane, thereby favoring magnetization components along the radial direction (see inset Figure 4g). This precession also results in a larger positive *m*_*z*_ component at the end of the pulse (Figure 4f), explaining the faster evolution of *m*_*z*_ observed under FL‐SOT compared to DL‐SOT in Figure 4g. Consequently, the final magnetic state under pure FL‐SOT is highly sensitive to the pulse duration since it determines the number of precessional cycles completed around the polarization axis.

## 4 Triggering Transition Between Different Types of Spin Textures With the Ultrafast Radial Current

For practical applications in QP‐based memory, controlled transitions between distinct *n*π QPs are essential. We theoretically propose bidirectional interconversion using a second laser pulse: a positive applied voltage (*V* > 0) converts an *n*π QP into an (*n* − 1)π QP (see Supporting Information S1: Figure S3), which for the case of a skyrmion (*n* = 1) corresponds to its erasure. Conversely, a negative voltage (*V* < 0) drives the transition from an *n*π QP to an (*n* + 1)π QP (see Supporting Information S1: Figure S4). To elucidate the mechanism underlying these transitions, Figure 5 presents the magnetic configurations for all four quasiparticle types 0.5 ps after laser excitation, under both positive and negative bias voltages.

**FIGURE 5:**
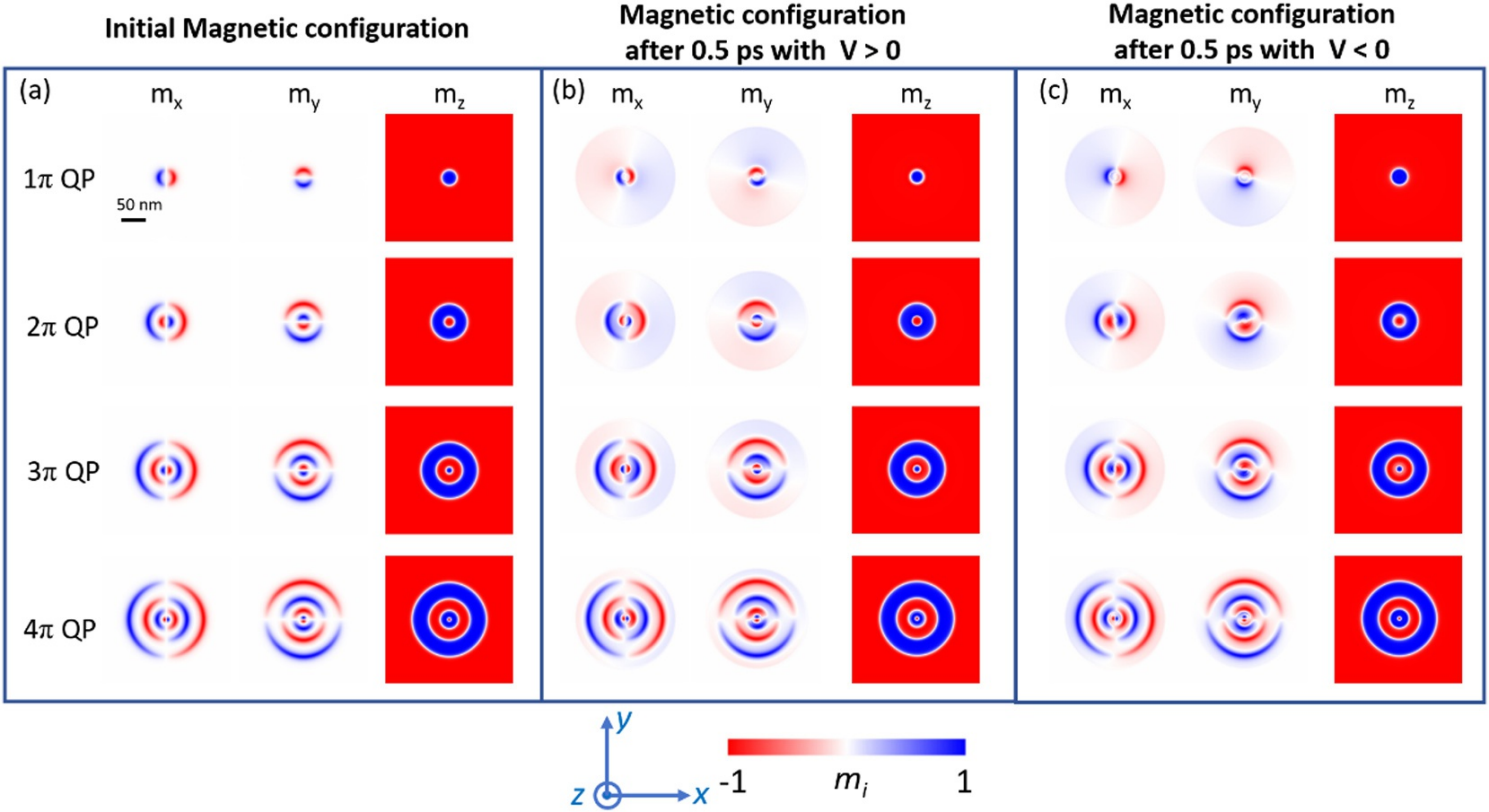
Laser effect on preexisting *n*π quasiparticles. Magnetic configuration (a) before and 0.5 ps after the beginning of the laser pulse with a (b) positive (*V* > 0) and a (c) negative voltage (*V* < 0).

Figure 5a reveals that the magnetization of the outermost domain wall for all quasiparticle types is initially aligned parallel to the radial unit vector $e_{r}$, a direct consequence of the chiral symmetry imposed by the DMI at equilibrium. However, under a positive applied voltage (Figure 5b), the photoinduced SOT generates a radial magnetization component that is antiparallel to the initial orientation at the QP boundary. As established in Figure 4d–e, this originates from the FL‐SOT, which drives precession around $e_{\theta }$ in a direction governed by the voltage sign. This antiparallel alignment causes all quasiparticles to contract. At high current densities, this compression can induce a topological transition to an (*n* − 1)π QP, as detailed in Supporting Information S1: Figure S3. Conversely, a negative applied voltage (Figure 5c) generates a SOT‐induced radial component parallel to the initial edge magnetization, which triggers the growth of the quasiparticle (see Supporting Information S1: Figure S5). At high enough current densities (Supporting Information S1: Figure S4), this expansion of the outermost domain wall of the quasiparticle is accompanied by with a strong effect of the laser at the center that triggers the topology change from an *n*π QP to an (*n* + 1)π QP.

## 5 Discussion

The efficient electrical generation and control of topological spin textures is a critical challenge for next‐generation spintronic memories. In this work, we theoretically propose an ultrafast method for generating and manipulating magnetic quasiparticles using a photoinduced radial current. This current reaches transient densities of 6.12 × 10^13^ A/m with an exceptionally low optical pump fluence of only 2.93 μJ/cm^2^. Although the current density is high, the resultant Joule heating is negligible (picojoule‐level) due to the picosecond duration of the current pulse and the high photoconductivity of the carrier‐rich LT‐GaAs layer. This energy efficiency represents a significant advantage over conventional skyrmion manipulation schemes, which often rely on thermal gradients from Joule heating [45], and even surpasses all‐optical switching techniques that require fluences on the order of mJ/cm^2^ [46, 47, 48, 49]. The mechanism enabling this low‐energy operation is the high built‐in electric field between the concentric Au electrodes [34, 50] (Supporting Information S1: Figure S1), which rapidly accelerates high‐mobility photogenerated carriers, yielding large current densities from minimal optical input. The two electrodes can be processed by the reported bench‐top method, combining with photolithography [51]. In practice, impedance mismatch from various factors such as material defects can cause the experimental spin current to deviate from simulated values. Since the spin–orbit torque magnitude dictates the quasiparticle order (Figure 2), this discrepancy may require an initial readjustment of the beam fluence to reliably generate each quasiparticle type.

Furthermore, our technique enables the generation of higher‐order *n*π quasiparticles, which we have previously shown to exhibit superior velocities. Unlike previous optical generation methods that required complex setups (e.g., tightly focused 4π configurations), our approach utilizes a simple optical focusing system. A present limitation is the material‐specific absorption of LT‐GaAs near 800 nm that hinders the use of other optical wavelength beams. Due to the diffraction‐limited, optical beams cannot be focused to a spot smaller than 0.5*λ*/NA, where *λ* is the wavelength and NA is the numerical aperture of the focus lens [52, 53]. Thanks to the development of plasmonics and near‐field optical technologies, beyond the diffraction limit has been studied for many years [54, 55]. To overcome the diffraction limit and focus the wavelength of 800 nm to the top of the rings of LT‐GaAs with a radius of 100 nm, a near‐field transducer, which is used in heat‐assisted magnetic recordings, can be selected to achieve efficient optical energy transfer [52, 56]. Near‐field transducers can efficiently concentrate 800 nm light to spots as small as 44 nm [57], making them ideal for coupling light into our sub‐100 nm device and paving the way for practical, scalable device integration. In addition, the research field of spin LED, which is to integrate GaAs semiconductor‐based LEDs with ferromagnets [58], makes it possible for GaAs deposition and the contact pads on the HM/FM layer. Although fabricating devices at the nanometric scale presents a barrier, our method can be implemented with larger devices. This adaptation allows for the generation of all four quasiparticle types, which will initially be oversized before contracting to equilibrium [19]. This workaround, however, results in increased power consumption.

## 6 Conclusions

In summary, we have theoretically proposed a novel mechanism for the ultrafast optical generation and control of topological spin textures. We achieved this by generating a picosecond‐scale radial photocurrent in a ring‐shaped LT‐GaAs layer (inner radius: 10 nm, outer radius: 100 nm) under a radial bias voltage. This current induces an azimuthally polarized SOT in an adjacent HM/FM bilayer, leading to the formation of stable whirling spin textures via the interfacial DMI. Through micromagnetic simulations, we showed that this unique torque geometry enables the ultrafast (< 50 ps) generation of all four target quasiparticle types (*n*π, *n* = 1–4). Crucially, the method also permits controlled transitions between these topological states by tuning the laser fluence and bias voltage. This combination of ultrafast speed, low energy consumption, and bidirectional control establishes our approach as a highly promising platform for future low‐power, high‐speed magnetic memory and logic technologies.

## Author Contributions

**Xiaoqiang Zhang:** conceptualization, investigation, writing – original draft, writing – review and editing, visualization, methodology, software, formal analysis, data curation, supervision. **Pierre Vallobra:** investigation, writing – original draft, writing – review and editing, visualization, methodology, software, formal analysis, data curation, supervision. **Yong Xu:** writing – review and editing, methodology. **Xiaoyang Lin:** writing – review and editing, supervision. **Weisheng Zhao:** writing – review and editing, supervision.

## Conflicts of Interest

The authors declare no conflicts of interest.

## Supporting information


Supporting Information S1

**Video S1:** Evolution of the magnetic configuration during the topological transition leading to the formation of a π quasiparticle.

**Video S2:** Evolution of the magnetic configuration during the topological transition leading to the formation of a 3π quasiparticle.

## Data Availability

The data that support the findings of this study are available on request from the corresponding author. The data are not publicly available due to privacy or ethical restrictions.
