# Supplementary material for: Photoinduced Picosecond‐Scale Radial Current for Generating and Converting Spin Textures
Source: Nanophotonics. 2026 Apr 4;15(7):e70060. doi: 10.1002/nap2.70060 (PMC13642615; doi:10.1002/nap2.70060)
Supplement: Supplementary file 1 [file NAP2-15-e70060-s002.docx]

**Supporting Materials:**

**Note 1. The distribution of the electric potential and the electric field in the LT-GaAs with a radially biased voltage**

Figure S1 shows the distribution of the electric potential and the electric field in the LT-GaAs with a radially biased voltage.

Figure S1. The distribution of the electric potential in the LT-GaAs with a radially biased voltage. The red arrows represent the direction of the inner built radial direction electric field and the black circles represent equal electric field intensity. (b) The distribution of the electric field in the LT-GaAs with a radially biased voltage.

**Note 2. Calculation the current density under different laser fluences and biased voltages**

Based on the Drude-Lorentz theory of carrier transport in semiconductors [1], the carrier density is time dependence and determined by the following equation [2]

$\frac{dn_{e,h}}{dt}=-\frac{n_{e,h}}{\tau_{e,h}}+G\left( t \right)$. (S1)

Here $n_{e,h}$ is the density of photoinduced carriers. $\tau_{e,h}$ is the carrier lifetime, which are 100 fs and 400 fs for the electron and hole, respectively. G(t) is the carrier generation rate under the pump of the laser, and it is

$G\left( t \right)=n_{0}e^{-\frac{\left( t-t_{0} \right)^{2}}{\tau^{2}}}$. (S2)

Here, *n*_0_ is the carrier generation density at the laser peak intensity, which is relate to the laser peak intensity *I*_0_, absorption *α*, optical frequency *v*, and it can be written as $n_{0}=\frac{\alpha I_{0}}{h\upsilon}\sim F_{0}$. The generated carriers will be accelerated by the inner built radial direction electric field, and their acceleration is given by

$\frac{dv_{e,h}}{dt}=-\frac{v_{e,h}}{\tau_{s}}+\frac{q_{e,h}}{m_{e,h}}E\left( r,t \right)$, (S3)

where $v_{e,h}$ is the average velocity of the carrier, $q_{e,h}$ is the charge of an electron (a hole), $m_{e,h}$ is the effective mass of the electron (hole), and $\tau_{s}$ is the momentum relaxation time. In Eq. (S2), *E* is the local electric field, which is smaller than the static electric field *E_d_* induced by the applied biased voltage, and they have the relationship as following

$E\left( r,t \right)=E_{d}\left( r \right)-\frac{P\left( t \right)}{g\epsilon}$, (S4)

where P is the polarization generated by the spatial separation of the electron and hole, $\epsilon$ is the dielectric constant of the LT-GaAs, which is 12.9, and *g* is the geometrical factor with value of *g* = 3. The polarization *P* has the following relationship

$\frac{dP}{dt}=-\frac{P}{\tau_{r}}+J\left( t \right)$. , (S5)

Here $\tau_{r}$ is the recombination time between an electron and hole. Then the density of the photoinduced current contributed by the generated electron and hole is

$J\left( t \right)=en_{h}v_{h}-en_{e}v_{e}$. (S6)

Figure S2 shows the photoinduced radial current density with arb. unit scale under different laser fluence and electric field strengths introduced by applying biased voltages. Here *F_0_* and *E_0_* are relative laser fluence and electric field strength.

Figure S2. Simulated the current density (CD) under different laser fluences and electric field strengths.

**Note 3. The transition between different spin textures by the ultrafast radial current**

As mentioned in the discussion of Fig.5, it is possible with a pulse to trigger a transition from a *n*π QP to a (*n*-1)π QP when applying a negative voltage. We observe that during the pulse each quasiparticle shrinks in size. It can also be noticed that the quasiparticles still shrink in size after the end of the pulse as the system is not in equilibrium, similarly to the excitation of a skyrmion breathing mode.

**Figure S3. Transition from nπ QP to (*n*-1)π QP.** Time evolution of the *z* component of the magnetization starting from the 4 types of quasiparticles a) during and b) after the pulse. The charge current densities are 8×*J*_0_(*r*,*t*), 6×*J*_0_(*r*,*t*), 6×*J*_0_(*r*,*t*) and 2×*J*_0_(*r*,*t*) for *n*= 1, 2, 3 and 4 respectively.

Similarly, it is possible with a pulse to trigger a transition from a nπ QP to a (*n*+1)π QP when applying a positive voltage. This process is a bit more complex since it involves both a spatial extension of the quasiparticle and a change of topology at the center of the beam. The spatial extension of the quasiparticle can be observed independently to a change of topology at lower current density, as observed in Figure S5. One can also notice that the absolute value of the voltage is much higher for a transition from a *n*π QP to a (*n*+1)π QP than to a (*n*-1)π QP. This can be explained by the fact that the shrinking of the quasiparticle is enough to also trigger a change of topology at the center due to the pressure exerted by most outside domain walls.

**Figure S4. Transition from *n*π QP to (*n*+1)π QP.** Time evolution of the *z* component of the magnetization starting from the 4 types of quasiparticles a) during and b) after the pulse. The charge current densities are -22×*J*_0_(*r*,*t*), -22×*J*_0_(*r*,*t*), -18×*J*_0_(*r*,*t*) and -22×*J*_0_(*r*,*t*) for *n* = 1, 2, 3 and 4 respectively.

**Figure S5. Growth of the *n*π QP at negative biased voltage.** Time evolution of the *z* component of the magnetization starting from the 4 types of quasiparticles a) during and b) after the pulse. The charge current density is of -16×*J*_0_(*r*,*t*) for all quasiparticles

**Reference:**

[1] M. Born and E. Wolf: Principles of Optics (Pergamon, New York, 1959).

[2] Z. Piao, M. TANI and K. Sakai, Carrier Dynamics and Terahertz Radiation in Photoconductive Antennas, Jpn. J. Appl. Phys. 39, 96 (2000).
